# Pyruvate Carboxylase Is Up-Regulated in Breast Cancer and Essential to Support Growth and Invasion of MDA-MB-231 Cells

**DOI:** 10.1371/journal.pone.0129848

**Published:** 2015-06-12

**Authors:** Phatchariya Phannasil, Chanitra Thuwajit, Malee Warnnissorn, John C. Wallace, Michael J. MacDonald, Sarawut Jitrapakdee

**Affiliations:** 1 Department of Biochemistry, Faculty of Science, Mahidol University, Bangkok, Thailand; 2 Department of Immunology, Faculty of Medicine, Siriraj Hospital, Mahidol University, Bangkok, Thailand; 3 Department of Pathology, Faculty of Medicine, Siriraj Hospital, Mahidol University, Bangkok, Thailand; 4 School of Molecular and Biomedical Sciences, University of Adelaide, Adelaide, SA5005, Australia; 5 Childrens Diabetes Center, University of Wisconsin School of Medicine and Public Health, Madison, WI, United States of America; University of Nebraska Medical Center, UNITED STATES

## Abstract

Pyruvate carboxylase (PC) is an anaplerotic enzyme that catalyzes the carboxylation of pyruvate to oxaloacetate, which is crucial for replenishing tricarboxylic acid cycle intermediates when they are used for biosynthetic purposes. We examined the expression of PC by immunohistochemistry of paraffin-embedded breast tissue sections of 57 breast cancer patients with different stages of cancer progression. PC was expressed in the cancerous areas of breast tissue at higher levels than in the non-cancerous areas. We also found statistical association between the levels of PC expression and tumor size and tumor stage (P < 0.05). The involvement of PC with these two parameters was further studied in four breast cancer cell lines with different metastatic potentials; i.e., MCF-7, SKBR3 (low metastasis), MDA-MB-435 (moderate metastasis) and MDA-MB-231 (high metastasis). The abundance of both PC mRNA and protein in MDA-MB-231 and MDA-MB-435 cells was 2-3-fold higher than that in MCF-7 and SKBR3 cells. siRNA-mediated knockdown of PC expression in MDA-MB-231 and MDA-MB-435 cells resulted in a 50% reduction of cell proliferation, migration and *in vitro* invasion ability, under both glutamine-dependent and glutamine-depleted conditions. Overexpression of PC in MCF-7 cells resulted in a 2-fold increase in their proliferation rate, migration and invasion abilities. Taken together the above results suggest that anaplerosis via PC is important for breast cancer cells to support their growth and motility.

## Introduction

As a result of over-stimulation by growth factor signaling, most tumors adapt their metabolism in order to accommodate both energy and structural component needs during rapid proliferation [1]. Unlike differentiated cells, regardless of the presence of oxygen, most tumors metabolize glucose via anaerobic glycolysis known as the ‘Warburg effect’ [2,3,4]. As the result of this metabolic shift, tumors consume large amounts of glucose, causing the accumulation of lactate. The enhanced glucose utilization via anaerobic glycolysis partly results from the over-expression of *c*-myc and hypoxia inducible factor-1α (HIF1α), which in turn stimulates expression of hexokinase II, lactate dehydrogenase (LDH), and the pyruvate kinase M2 isoform (PK-M2) that enhance glycolysis, and pyruvate dehydrogenase kinase (PDK) that can inhibit the oxidative decarboxylation of pyruvate by phosphorylation of pyruvate dehydrogenase. This phenomenon results in channeling of pyruvate into anaerobic glycolysis and limits its entry into mitochondria for oxidative phosphorylation and also shifts mitochondrial metabolism towards biosynthetic purposes [5,6,7,8]. Anaplerosis via pyruvate carboxylation and glutamate synthesis from glutamine (glutaminolysis) are the two major biochemical reactions which replenish TCA cycle intermediates withdrawn for biosynthetic pathways [9]. Furthermore, up-regulation of expression by c-*myc* of glutaminase, an enzyme which converts glutamine to glutamate is also observed in many tumors [10,11,12,13]. This high demand for glutamine is a hallmark of most tumors and was proposed as a target of cancer treatment. While growing evidence have now indicated that glutaminolysis is crucial for many cancers, limited information is available regarding the importance of pyruvate carboxylation via pyruvate carboxylase (PC) in cancers.

Fan *et al*. [14] have shown that over-expression of PC mRNA and protein was detected in solid cancerous lung tissue. ^13^C Isotopomer-based metabolomic analysis reveals that pyruvate carboxylation flux is increased in the cancerous lung tissue compared to the paired non-cancerous lung tissues, suggesting that anaplerosis via pyruvate carboxylation is essential to fulfill the high anabolic demand of lung tumor during the establishment of primary tumorigenesis. By infusing ^13^C-labeled glucose or ^15^N-labeled glutamine in the patients with early-stage non-small cell lung cancer before tissue resection, the same group of investigator showed that the labeled glucose enters mitochondrial metabolism via pyruvate carboxylation while this not the case for ^15^N-glutamine, indicating that anapleorosis via PC is the preferred route in non-small cell lung cancer [15]. Furthermore suppression of PC expression in non-small cell lung cancer cell lines resulted in the retardation of cell growth primarily due to the disruption of biosynthesis of lipid and nucleotide and imbalance of glutathione metabolism [15].

PC is also involved in brain tumor progression. Interestingly, many low grade glioblastomas possess a defect in the cytosolic isocitrate dehydrogenase (IDH1) which functions in converting isocitrate to α-ketoglutarate concomitant with the production of NADPH. The α-ketoglutarate once formed in the cytoplasm by IDH1 can re-enter mitochondria and be used for various biosynthetic pathways [16]. Mutation of IDH1 is predicted to impair mitochondrial metabolism in many cancers [17]. Izquierdo-Garcia *et al* [18] have recently engineered astrocytes to metabolically mimic low grade glioblastomas by expressing mutated IDH1 and found that these astrocytes bearing an IDH1 mutation bypass the IDH1 defect by up-regulating pyruvate carboxylation. In supporting this finding, Cheng *et al*. [19] have demonstrated that PC is highly abundant in some glioblastoma cell lines, and is required to support its growth under glutamine-independent conditions. The above evidence highlights the roles of PC in supporting the growth of lung and brain tumors.

Although PC has been shown to be highly abundant in these two types of tumor and appears to support growth of glioblastoma under glutamine-independent growth, it remains unclear whether PC is highly expressed in other types of cancer and is also required to support other aggressive phenotypes. Here we show that PC is overexpressed in breast cancer tissues, and that the level of expression is correlated with tumor size and stage. We also demonstrate that suppression of PC expression in a highly metastasized cell line, MDA-MB-231 markedly reduces its proliferation, migration and *in vitro* invasion ability, indicating the importance of PC to support growth and invasion of breast cancer.

## Materials and Methods

### Cell culture

Human breast cancer cell lines, MCF-7 (ATCC:HTB22) [20] and MDA-MB-231(ATCC: HTB26) [21], MDA-MB-435 (ATCC:HTB129) and SKBR3 (ATCC: HTB-30) were grown in Dulbecco’s modified Eagle’s medium (DMEM) (Gibco) supplemented with 10% (v/v) fetal bovine serum (FBS). The cells were maintained at 37^°^C with 5% CO_2_. The glutamine-independent MDA-MB-231 cell line was established by progressive depletion of glutamine in the culture medium from 4 mM to 0 mM. In brief, cells were grown in DMEM supplemented with 2 mM glutamine for 2 weeks, 1 mM for 2 weeks, 0.5 mM for 2 weeks and no glutamine, respectively. After 1 month of growing this cell line in the absence of glutamine, it was used for subsequent experiments.

### siRNA transfection of and overexpression of PC

6 x 10^5^cells of MDA-MB-231 cells or 3.5 x 10^5^cells of MDA-MB-435 were plated in 35-mm dish containing 2 ml of DMEM supplemented with 10% (v/v) FBS and maintained at 37°C with 5% CO_2_ for 24 h. 50 pmole (25 nM) or 100 pmole (50 nM) of siRNA targeted to human PC (Cat.no. 4390824, Ambion) were transfected to MDA-MB-231 or MDA-MB-435, respectively using Lipofectamine 2000 transfection reagent (Invitrogen) in the Optimem-reduced serum medium (Invitrogen). Same amounts of scrambled control siRNA were also transfected to both cell lines. The transfected cells were maintained in 2 ml complete medium for 2 days. The cells were subsequently harvested for RT-PCR and Western blot analyses.

MCF-7 cells overexpressing PC were generated by transfection of plasmid encoding human PC (pEF-PC) [22]. In brief, 2 x 10^5^ cells of MCF-7 were plated in 2 ml complete DMEM medium in 35 mm-dish 24 h before transfecting with 4 μg of pEF-PC plasmid. Upon 48 h post-transfection, the stable MCF-7 cells overexpressing PC cells were selected with 0.5 μg/mL puromycin for one week. The stable lines were expanded for another week before proliferation, migration and invasion assays were performed.

### Reverse transcription polymerase chain Reaction (RT-PCR)

Total RNA was extracted from cells using TRIzolReagent (Gibco) following the manufacturer’s instructions. Initially the random hexamers-primed RNA was carried out in a 10 μl-reaction mixture containing 2 μg of total RNA and 200 ng of random hexamers (Promega) at 70^°^C for 5 min, before being chilled at 4^°^C. Reverse transcription was initiated by adding 10 μl of mixture containing 1xImProm-II reaction buffer, 3 mM MgCl_2_, 0.5 mM dNTP mix and 160 units of ImProm-II reverse transcriptase (Promega), to the primed-RNA mixture and the reaction was incubated at 25^°^C for 5 min, 42^°^C for 60 min and 70^°^C for 15 min, respectively. The cDNA was stored at -20^°^C until used.

### Quantitative real-time PCR

Quantitative real time PCR was performed in a 12 μl-reaction mixture containing 6 μl of 2xKAPA probe Fast qPCRmaster mix Universal (KAPA Biosystems), 2 μl of cDNA, 1 μM of forward and reverse primers and 0.5 μM of fluorogenic probe as described previously [23]. The thermal profiles consisted of initial incubation at 50^°^C for 2 min and 95^°^C for 10 min followed by 40 cycles of denaturation at 95^°^C for 15 sec and annealing/extension at 60^°^C for 1 min in Mx3000P Q- PCR system (Agilent Technologies). To identify the PC mRNA isoforms expressed in breast cancer cell lines, quantitative real time PCR was performed in a 20 μl-reaction mixture containing 10 μl of 2x KAPA SYBR FAST qPCR Master mix universal (KAPA Biosystems), 200 nM each of specific forward primer for detecting each isoform of PC mRNA and reverse primers [24], and 2 μl of cDNA. The thermal profiles consisted of initial incubation at 95^°^C for 10 min followed by 40 cycles of denaturation at 95^°^C for 30 sec, annealing at 60^°^C for 30 sec and extension at 72^°^C for 30 sec in Mx 3000P Q- PCR systems (Agilent Technologies). Expression of PC mRNA was normalized with that of 18s ribosomal RNA gene, and was shown as the relative gene expression. Fold change was calculated using the comparative C_T_ method (∆∆C_T_ method) [25].

### Western blot analysis

MCF-7, MDA-MB-231 and MDA-MB435 cells grown in T75 cm^2^ flask were trypsinized with 0.05% (v/v) trypsin-EDTA. The detached cells were centrifuged at 3,000 xg for 5 min, cell pellet was re-suspended in 150 μl of RIPA buffer (50 mM Tris-HCl pH 7.4, 150 mM NaCl, 1 mM EDTA, 0.25% sodium deoxycholate, 1% (v/v) NP-40 and 1x protease inhibitor cocktail (Roche). 70 μg of protein lysate were subjected to discontinuous SDS-PAGE [26] under reducing conditions. The proteins were transferred to a polyvinylidenedifluoride (PVDF) membrane by Semi-Dry Transfer Cell (BIO-RAD) for 1.5 h. The membrane was incubated in 15 ml of blocking solution [5% (w/v) skim milk in 1% (v/v) Tween 20 in PBS-T] at 4^°^C overnight. For detecting PC protein, the blot was incubated with1:5,000 dilution of rabbit anti-chicken PC polyclonal antibody [27] for 2 h. The blot was briefly washed in PBS-T before incubating with 1:5,000 dilution of goat anti-rabbit IgG conjugated with horseradish peroxidase (HRP) (DAKO) for 1 h. For detection of β-actin, mouse anti-actin monoclonal antibody (sc-8432) (Santa Cruz) and sheep anti-mouse IgG conjugated with HRP (GE healthcare) were used for primary and secondary antibodies, respectively. The immunoreactive bands were detected using an enhanced chemiluminescence substrate (Perkin Elmer). The images were captured using Chemiluminescence Imaging System (Syngene).

### Proliferation assay

Cell proliferation was determined by counting the viable cells for 7 days. At 48 h post-transfection, the 1 x 10^5^ cells of MDA-MB-231, MDA-MB-435 or MCF-7 overexpressing PC were plated into 35 mm dishes and grown in the absence or presence of 4 mM glutamine for 1, 2, 3, 4, 5, 6 and 7 days, at 37°C in CO_2_ incubator. At each time point, the cells were trypsinized, stained with 0.4% trypan blue and counted under a microscope. The results are presented as means + standard deviations of two independent experiments.

### Wound healing assay

1.5 x 10^5^ cells of MDA-MB-231, MDA-MB-435 and MCF-7 overexpressing PC were replated in 24-well plate in DMEM containing no serum overnight before the artificial wound was generated by scratching the monolayer with a pipette tip. The wound’s closure (width) of the PC knockdown at 0 and 48 h was measured and shown as the mean + standard deviation of that of the scrambled control which was arbitrarily set as 100%.

### *In vitro* invasion assay

*In vitro* invasion assays were performed by plating 1.2 x 10^5^ of MDA-MD-231, MDA, MDA-MB-435 or MCF-7 cells in 200 μl of serum-free medium containing 4 mM or 0 mM glutamine into the upper chamber of Transwell (6.5-mm diameter polyvinylpyrrolidone-free polycarbonate filter of 8-μm pore size) (Corning, NY, USA) which was pre-coated with 20 μg Matrigel (BD Biosciences) while the lower chamber contained medium supplemented with 10% (v/v) FBS for 4 h (for MDA-MB-231) or with 20% FBS for 24 h (for MCF7 and MDA-MB-435) at 37^°^C. The non-invaded cells in the upper compartment were removed and the chamber was washed twice with 1x PBS. The cells that had invaded through the matrix were fixed with 4% (v/v) paraformaldehyde in 1x PBS for 20 min and stained with 0.5% crystal violet in 25% (v/v) methanol overnight, followed by two washes with tap water. Finally, the invaded cells were counted under a microscope and the percentage of invasion was compared with that of the scrambled control cell which was arbitrarily set as 100%.

### Immunohistochemistry (IHC)

Fifty-seven paraffin-embedded breast cancer tissue sections were collected under the protocols approved by Siriraj Institute Revision Board, Faculty of Medicine Siriraj Hospital, Mahidol University (COA no. Si 230/2014), and all clinical investigation were conducted according to the principles expressed in the Declaration of Helsinki. The written informed consent was obtained from each participant who enrolled in the study. Those breast tissues were diagnosed invasive ductal carcinoma. Firstly, the antigen was retrieved by incubation with 10 mM citrate buffer pH 6 at 95^°^C for 1 h. The sections were blocked with 2% (w/v) BSA for 20 min before incubating with 1:1,000 dilution of anti-chicken PC antibody [27] at room temperature overnight. Excess primary antibody was removed by washing with 1x PBS for 10 min before incubating with specific anti-rabbit EnVision+system-HRP labelled polymer (DAKO) at room temperature for 30 min. The secondary antibody was washed with 1x PBS for 10 min before 0.05% (w/v) 3,3’-diaminobenzidine (DAB) solution was applied to the sections and incubated at room temperate for 5 min. The sections were counter-stained with Mayer’s hematoxylin, dipped in 1% (w/v) lithium carbonate and washed with tap water for 5 min. Finally, the sections were mounted with Permount and examined under the microscope. PC expression was semi-quantitatively scored on the basis of percentage of PC-positive cells and the immunostaining intensity. Grading for the percentage of PC-positive cancer cells were as follows: 1 for 1–25%; 2 for 26–50%; 3 for 51–75% and 4 for 76–100%. The intensities of PC staining in cancer tissues were as follows: 0, unstained; 1, slightly; 2, intermediate and 3, strongest staining. The interpretation of PC expression was performed by the scores of the percent positive cells (1–4) multiplied by the scores of staining intensity (0–3) to reach the total final immunohistochemistry (IHC) score of 0–12. The results were then categorized as follows; low expression, IHC score ≤ 6; and high expression, IHC score > 6. All samples were anonymous and independently scored by two investigators of whom one is a pathologist.

### Statistical analysis

All values were expressed as mean ± standard deviations. The statistical analysis was performed using Student’s t-test, two way anova and univariate analysis where * *P* < 0.05, ** *P* < 0.01 and *** *P* value < 0.001.

## Results

### PC was overexpressed in breast cancer tissues and correlated with tumor size and late stage of tumor progression

**Fig 1:**
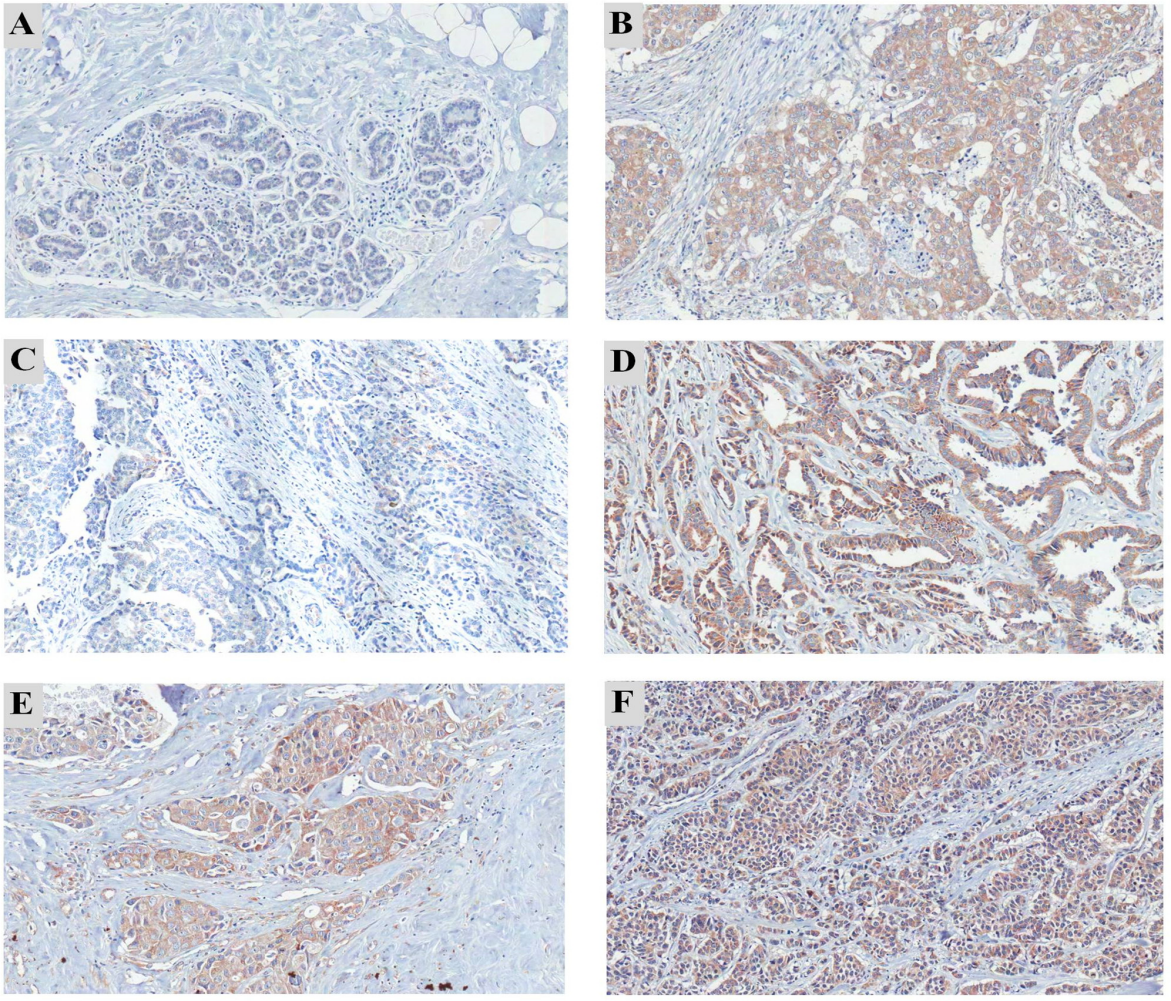
Immunohistochemistry staining of PC in paraffin-embedded breast tissue sections of patients with various stages of breast cancer. (A) Normal adjacent area of breast tissue showing weak staining of PC compared to strong staining in the cancerous area (B) of the same tissue. The representative samples showing different expression levels of PC in different stages of breast cancer: (C) stage 1, (D) stage 2, (E) stage 3 and (F) stage 4. Original magnification, 100x.

A previous study has reported the overexpression of PC in solid lung tumor tissue [14]. To examine whether this was the case for breast cancer, we performed immunohistochemistry (IHC) staining using an anti-PC antibody on breast tissue sections collected from 57 patients who had stages I-IV of breast cancer using anti-PC antibody. In contrast to the non-cancerous area of the tissue sections, (Fig 1A), PC was highly expressed in the cancerous areas of the breast tissues (Fig 1B). The expression levels of PC expression also varied in different stages of cancer (Fig 1C–1F). Expression of PC in stromal fibroblasts and infiltrating immune cells was rarely observed.

PC expression was detected in breast tissues of most cancer patients (96% of the total studied cases) except two cases which were stage I. Overall, 72% of breast cancer tissues showed a low expression level of PC whereas 28% had a high expression level of PC (Table 1). Based on grouping the patients into early stage without distant metastasis (stages I-III) and late stage with distant metastasis (stage IV), 67% (4 in 6 cases) of breast cancer with distant metastasis showed a high expression level of PC. In contrast, only 24% (12 in 51 cases) of patients without metastasis had a high level of PC expression. Univariate analysis showed a significant correlation between PC expression level and stage IV (P = 0.046). Interestingly, PC expression showed a significant correlation with the tumor size (Table 1) (P = 0.033) i.e., PC was poorly expressed in most tumor cases with small volume (<4 cm^3^) (86%, 25/29). The other clinicopathological parameters including histological type, invasion, estrogen receptor (ER), progesterone receptor (PR) and HER2 expression did not show statistical associations with PC expression.

**Table 1:** Univariate analysis of expression level of PC in breast tissues and clinicopathological parameters.

Variable (Total cases)		PC expression (IHC score)		P-value
	No of case	Low (< 6)	High (> 6)	
Tumor volume (cm^3^) (52)				
< 4	28	24	4	0.033*
> 4	24	14	10	
Tumor staging (57)				
I	18	15	3	0.225
II	22	17	5	0.555
III	11	7	4	0.482
IV	6	2	4	0.046*
Histological type (56)				
Well-differentiated	7	6	1	0.661
Moderately-differentiated	30	20	10	0.365
Poorly-differentiated	19	15	4	0.543
Invasion (57)				
Absence	24	18	6	0.769
Presence	33	23	10	
ER (57)				
High (4)	38	29	9	0.356
Low (0–3+)	19	12	7	
PR (57)				
High (4)	17	14	3	0.342
Low (0–3+)	40	27	13	
HER2 (57)				
Negative	37	27	10	1.00
Positive	20	14	6	

### PC is highly abundant in metastasized breast cell lines, MDA-MB-231 and MDA-MB-435

**Fig 2:**
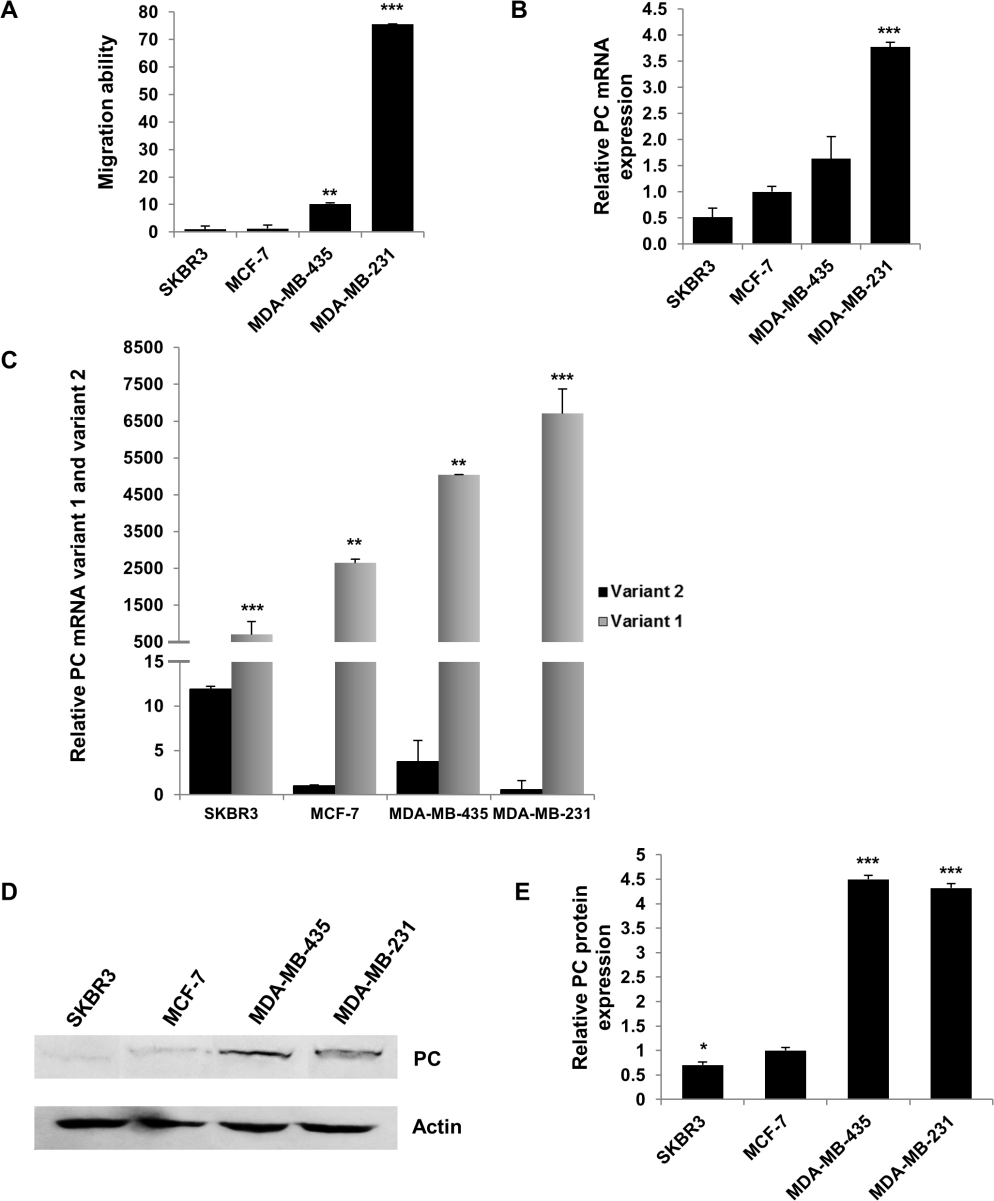
Migration ability of various breast cancer cell lines and the expression levels of PC in these cell lines. **A**, Migration ability of SKBR3, MCF-7, MDA-MB-435 and MDA-MB-231. **B**, Q-PCR analysis of PC mRNA expression in the above cell lines. The expression of PC was normalized with the expression of 18s rRNA gene and shown as the relative gene expression. The relative PC expression in MCF-7 was arbitrarily set as 1. **C**, Real time PCR analysis of PC mRNA variant 1 and 2 expression in the above cell lines. The expression of PC was normalized with the expression of 18s rRNA gene and shown as the relative gene expression. The expression of the relative PC mRNA variants in MCF-7 was arbitrarily set as 1. **D**, Western blot analysis of PC protein in the above cell lines. The blot was also probed with anti-actin antibody to serve as loading control. **E**, The immunoreactive band intensity of PC in D was quantitated and normalized with that of the β-actin and shown as the relative PC expression. The statistical analysis was conducted using student’s t-test where *P < 0.05, **P < 0.01, ***P < 0.001.

Because the degree of PC expression showed statistical association with tumor size and stage, we hypothesized that PC was required to support tumor growth and invasion of breast cancer cells. We examined the above hypothesis by investigating the expression levels of PC in breast cancer cell lines with different degrees of motility, namely, MCF-7, SKBR3, MDA-MB-435 and MDA-MB-231. As shown in Fig 2A, the motilities of MDA-MB-435 and MDA-MB-231 are 10-fold and 75-fold higher than those of MCF-7 and SKBR3 cells, respectively. Consistent with the motility phenotype, MCF-7 and SKBR3 cell lines possess low expression level of PC mRNA while MDA-MD-231 and MDA-MB-435 cell lines express PC mRNA 4-fold and 2-fold higher than MCF-7 (Fig 2B). The two alternative PC mRNA isoforms, namely variant 1 and variant 2 which differ in their 5’-untranslated regions, are differentially transcribed from two alternative promoters, the distal and the proximal promoters, respectively [24]. To examine which of these two PC mRNA isoforms was up-regulated in MDA-MB-231 and MDA-MB-435 cells, quantitative real-time PCR using primers specific for variants 1 and 2 was performed. As shown in Fig 2C, expression of variant 1 was up-regulated in all cell lines however both MDA-MB-231 and MDA-MB-431 possessed expression of variant 1 more than MCF-7 and SKBR3.

Western blot analysis of PC protein of these four cell lines was also consistent with their motility phenotype and PC mRNA expression i.e. the abundance of PC in MDA-MB-231 and MDA-MB-435 are 4.5-fold higher than in MCF-7 and SKBR3 (Fig 2D and 2E).

### Suppression of PC expression lowers growth, migration and invasion ability of highly metastasized breast cancer cell lines

**Fig 3:**
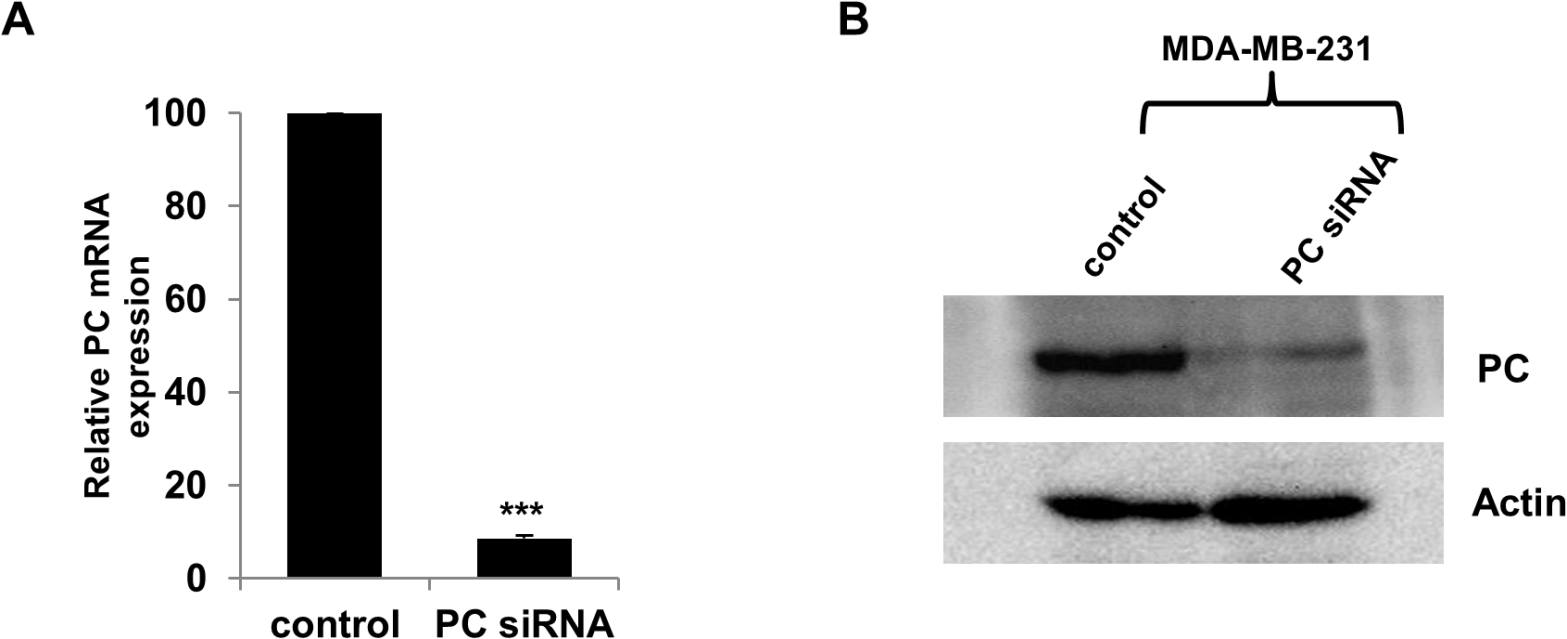
siRNA-mediated suppression of PC expression in MDA-MB-231 cell line. Real time PCR analysis of PC mRNA expression in MDA-MB-231 cells transfected with scrambled control (Control) or PC siRNA. The PC mRNA level was determined by Q-PCR at 48 h post-transfection (A). Western blot analysis of PC protein in the PC knockdown MDA-MB-231 and the scrambled control (B). The statistical analysis was conducted using student’s t-test ***P ≤ 0.001.

**Fig 4:**
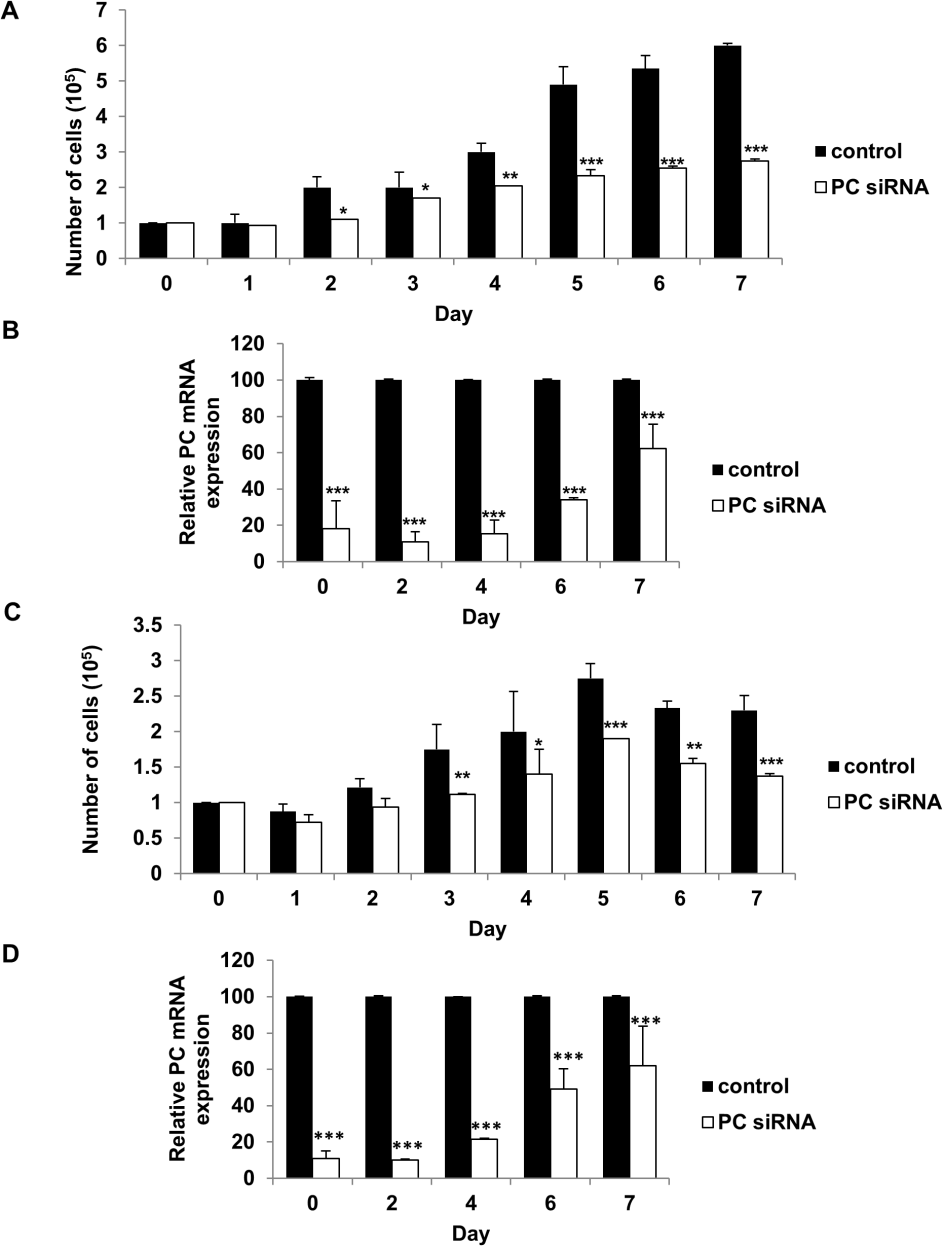
Suppression of PC expression in MDA-MB-231 retarded proliferation both in glutamine-nourished and glutamine-depleted conditions. MDA-MB-231 cells were transiently transfected with PC or scrambled control siRNAs. At 48 h post transfection cells were trypsinized, re-plated and grown in the presence of 0 mM or 4 mM glutamine for 7 days. The proliferation rate of the PC knocked down (PC siRNA) and the control MDA-MB-231 cell lines (Control) grown in the medium containing 4 mM (A) or 0 mM (C) glutamine. The relative expression of PC mRNA in the knocked down MDA-MB-231 cells grown in the presence of 4 mM (B) or 0 mM (D) glutamine throughout the assay. The results are means obtained from two independent experiments, each in triplicate. The statistical analysis was conducted using ANOVA test where *P < 0.05, **P < 0.01, ***P < 0.001.

**Fig 5:**
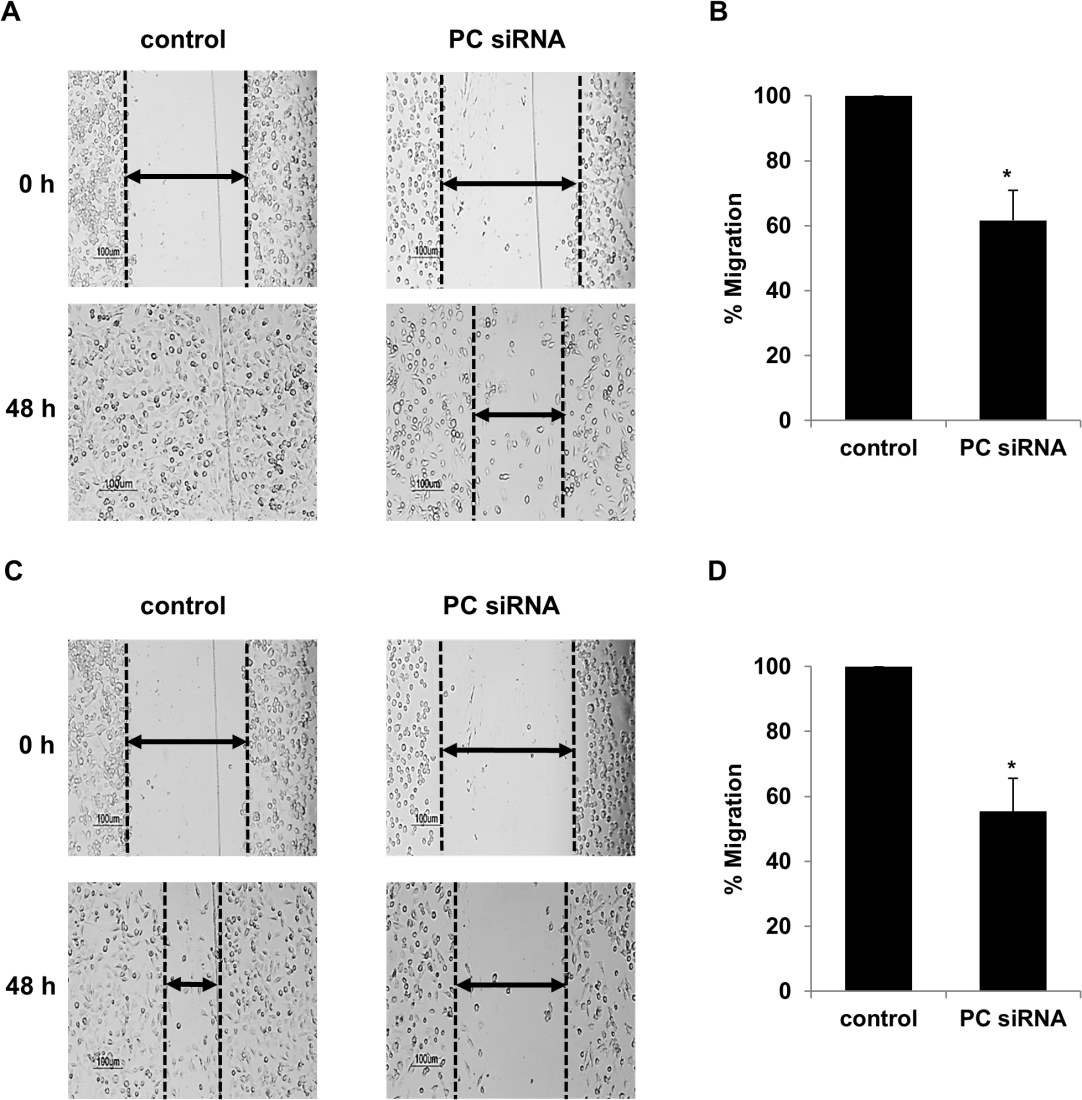
Suppression of PC expression in MDA-MB-231 cells reduced migration. Representative images of wound-healing assays. MDA-MB-231 cells were transiently transfected with PC or scrambled control siRNAs. At 48 h post transfection, wound-healing assays were performed as described in the materials and methods. (A, C) Representative images of the PC knockdown (PC siRNA) or scrambled control cells (Control) migrated across the wound areas in the presence of 4 mM (A) or absence of glutamine (C). The wound’s closure (width) of the PC knockdown was measured and shown as the means + standard deviation of that of the scrambled control which was arbitrarily set as 100% (B, D). The results were obtained from two independent experiments, each in triplicate. The statistical analysis was conducted using student’s t-test where *P < 0.05.

We next examined whether overexpression of PC in the MDA-MB-231 cell line was necessary to support its growth and invasion ability. We suppressed expression of PC in this cell line by siRNA and assessed the phenotypes of the knockdown cells. As shown in Fig 3A and 3B, suppression of PC expression in MDA-MB-231 resulted in 90% and 80% decreases in PC mRNA and PC protein levels, respectively. PC knockdown MDA-MB-231 cell line did not affect proliferation in the first day but showed a 50% reduction in growth by day 2 (Fig 4A). Similar degrees of reduction were observed until day 7. Real-time PCR analysis also confirmed that the retarded proliferation rate of this cell line was accompanied by suppression of PC mRNA throughout day 7 (Fig 4B). Because suppression of PC expression did not completely inhibit the cell proliferation rate, the ability of the PC knockdown MDA-MB-231 cells to grow in the complete growth medium may have resulted from the compensation of anaplerosis via glutaminolysis. To examine whether this latter pathway contributes to the survival of the PC knockdown cells, the glutamine-independent PC deficient MDA-MB-231 cell line was generated. In the absence of glutamine supplementation in the medium, any anaplerotic reaction would rely exclusively on a PC-catalyzed reaction. We generated the glutamine-independent MDA-MB-231 cell line (Gln^-^) by gradually depleting glutamine from the culture medium before transfecting this cell line with PCsiRNA so that the phenotype of this cell line became glutamine-independent and PC deficient (Gln^-^/PC^-^-MDA-MB-231). The Gln^-^/PC^-^-MDA-MB-231cells grown in the glutamine-free medium showed a growth rate similar to the control cell line in the first two days but showed approximately 30–40% reduction of cell proliferation from day 3 until day 7 (Fig 4C). Real time PCR analysis also confirmed that the retarded proliferation rate of this cell line was accompanied by suppression of PC mRNA throughout day 7 (Fig 4D). As the levels of PC expression were significantly correlated with the stages of cancer progression, we hypothesized that PC was involved in the aggressive phenotypes of breast cancer cells, particularly migration and invasion. We investigated whether PC was required to support migration and invasion ability of MDA-MB-231 cells. We first examined the ability of the PC knockdown MDA-MB-231 cells to migrate across a wound. As shown in Fig 5A and 5B, the PC knockdown cells exhibited a 40% reduction of migration across the wound compared to the scrambled control. A similar degree of reduction was observed from the glutamine-independent PC knockdown cell line (Fig 5C and 5D).

**Fig 6:**
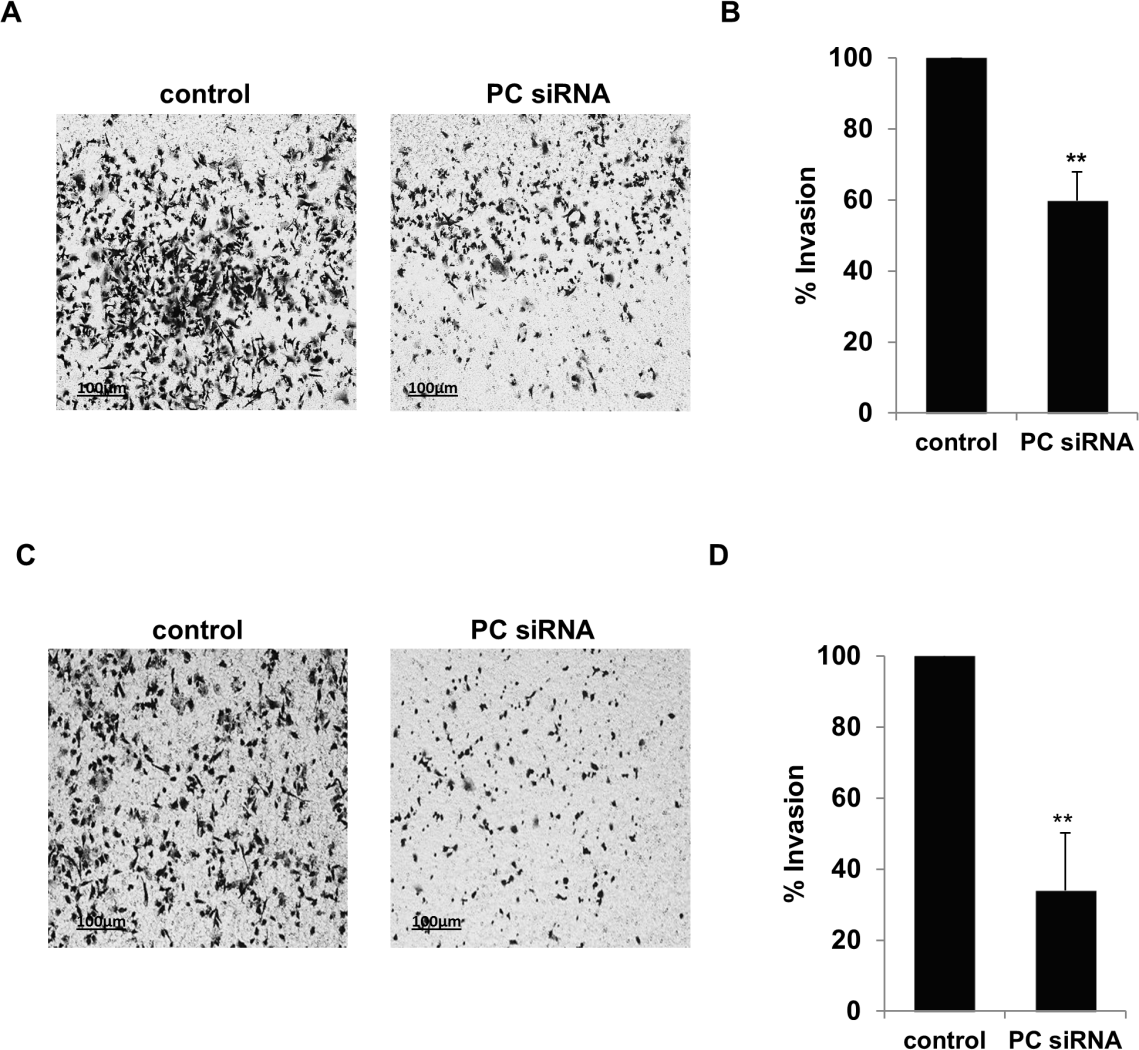
Suppression of PC expression in MDA-MB-231 lowers invasion ability. MDA-MB-231 cells were transiently transfected with PC or scrambled control siRNAs. At 48 h post transfection, an *in vitro* invasion assay was performed for 4 h in the presence of 4 mM (A) or 0 mM (C) glutamine. The number of PC siRNA-transfected cells that invaded the transwell coated with Matrigel was counted in 5 different fields and shown as means + standard deviation in comparison with that of the scrambled control which was arbitrarily set as 100% (B, D). The results were obtained from three independent experiments, each done in duplicate. The statistical analysis was conducted using student’s t-test where ** *P* < 0.01.

We next examined the ability of the knockdown cells to invade through an extracellular matrix by performing an *in vitro* invasion assay using transwell coated with Matrigel. As shown in Fig 6A and 6B, the PC knockdown cells showed a 40% reduction of their invasion ability. However, the reduced invasion ability was more pronounced (60%) in the glutamine-independent knockdown MDA-MB-231 cells (Fig 6C and 6D).

**Fig 7:**
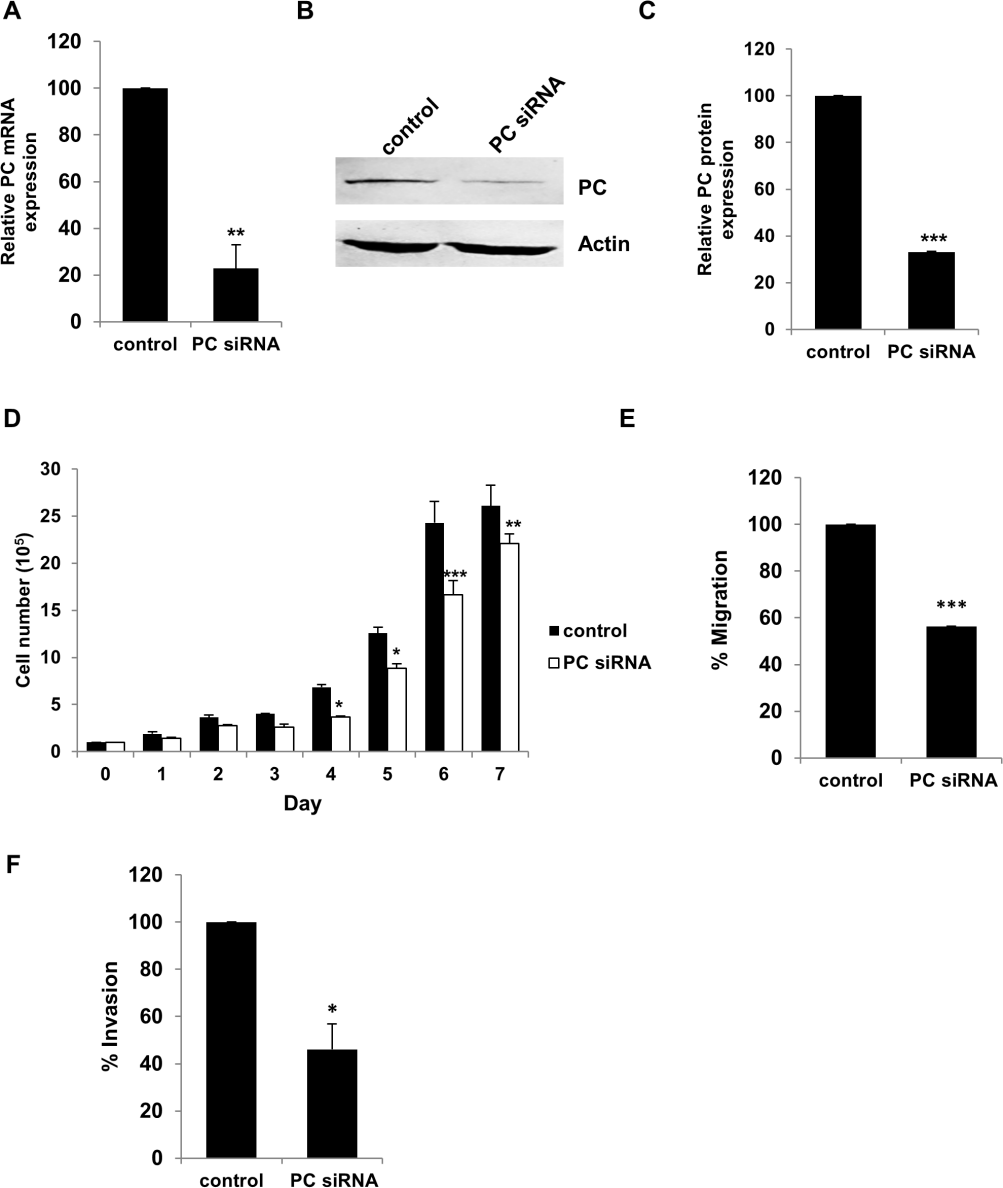
Suppression of PC expression in MDA-MB-435 cells reduces their proliferation, migration and invasion. PC mRNA expression in the knockdown MDA-MB-435 was quantitated by Q-PCR (A). Western blot analysis of MDA-MB-435 cells transfected with PC siRNA (PC siRNA) or scrambled control (Control) (B). The band intensity of PC in B was quantitated and normalized with β-actin band and expressed as relative PC expression (C). Proliferation assay (D), migration assay (E) and invasion assay (F) of the PC knockdown MDA-MB-435 cells. The statistical analyses in B, C, E and F were conducted using student’s t-test while in D was conducted using ANOVA test. *P < 0.05, **P < 0.01, ***P < 0.001.

We also performed similar experiments in MDA-MB-435 cells which also bear a high level of PC protein although its migration ability is less than MDA-MB-231. Suppression of PC mRNA expression by 80% (Fig 7A) resulted in 70% down-regulation of PC protein (Fig 7B and 7C). The PC knockdown MDA-MD-435 cells showed retarded growth rates at day 4 onwards (Fig 7D). Suppression of PC was also associated with 40% reduction of cell migration (Fig 7E) and 50% reduction of invasion ability (Fig 7F). Similar results were obtained when the knockdown cells were grown in the absence of glutamine (data not shown).

### Overexpression of PC in MCF-7 cells increases their proliferation and *in vitro* invasion

**Fig 8:**
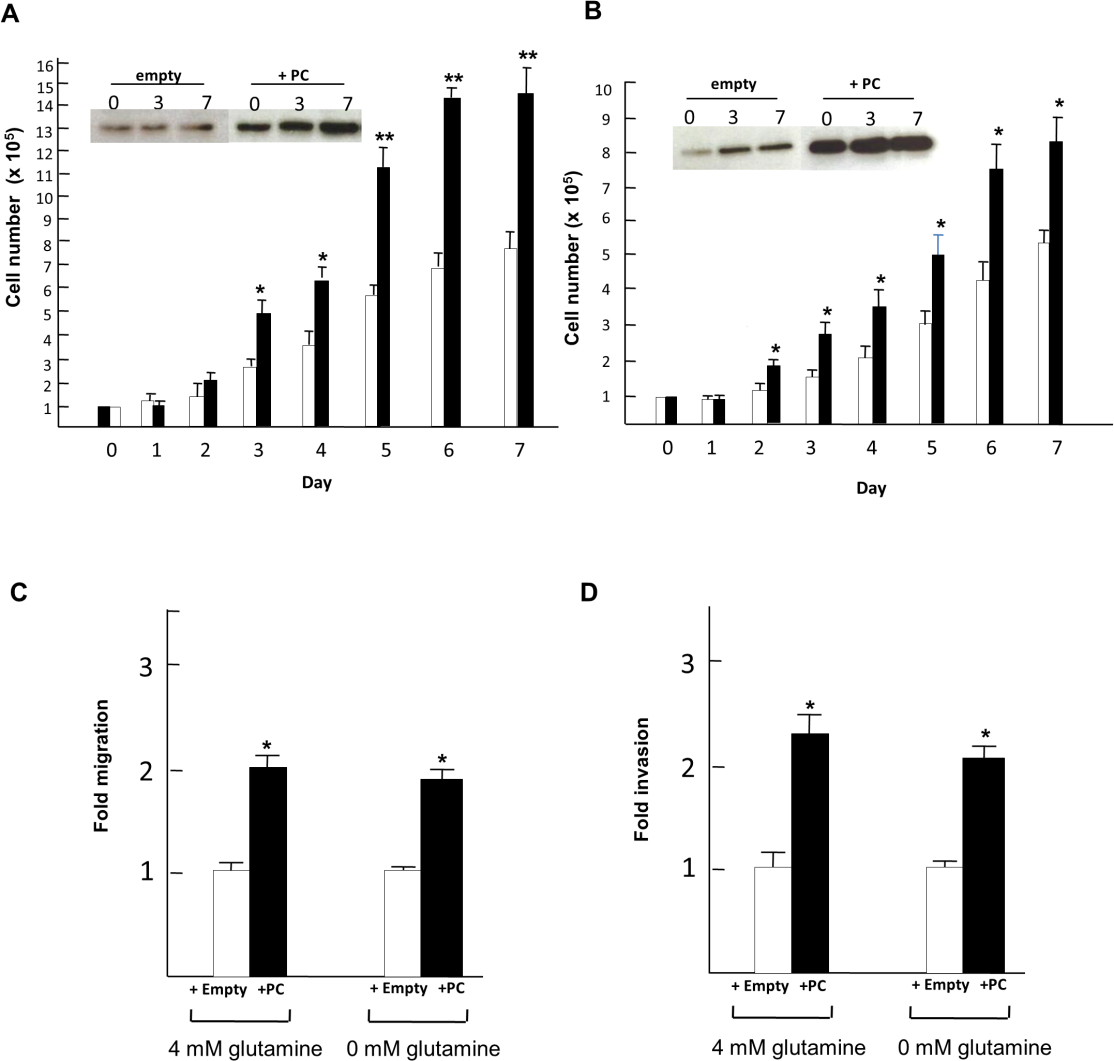
Overexpression of PC in MCF-7 cells increases their proliferation, migration and invasion. Proliferation rate of MCF-7 cells overexpressing PC grown in the medium containing 4 mM (A) or 0 mM glutamine (B). Insets in A and B are the Western blot analysis of MCF-7 cells transfected with empty vector (+empty, white bars) or with overexpressed PC (+PC, black bars) at days 0, 3 and 7. MCF-7 cells overexpressing PC grown in the presence or absence of glutamine were also subjected to migration (C) and invasion (D) assays. The statistical analysis was conducted using student’s t-test where * *P* < 0.05, ** *P* < 0.01.

To confirm whether overexpression of PC in breast cancer cells with low metastatic ability would increase their proliferation rate and invasion ability, we generated stable MCF-7 cell overexpressing PC. As shown in Fig 8A, the proliferation rate of MCF-7 cells at day 3 onward of MCF-7 with overexpressed PC was 2-fold higher than the MCF-7 cell line transfected with an empty vector. Similar results were observed when the MCF-7 cell line with overexpressed PC was grown in glutamine-depleted medium (Fig 8B). It is noted that the level of endogenous PC was slightly increased in the MCF-7 cell line harboring empty vector when these cells were grown in the absence of glutamine, suggesting a compensatory increase of PC expression in response to the deprivation of glutamine. MCF-7 cells with overexpressed PC also showed 2-fold and 2.5-fold increases in the migration and invasion ability, respectively. Similar results were obtained when the cells were assayed under glutamine-depleted medium (Fig 8C and 8D).

## Discussion

Anaplerotic reactions are important to replenish the levels of TCA cycle intermediates upon their removal for biosynthetic purposes [9,28]. Anaplerosis via glutaminolysis is well known to be important in many tumors as they rely on this reaction to provide precursors needed for the syntheses of structural components, i.e. membrane lipids and nucleic acids. Disruption of glutamine supply to many tumors is proposed to be one means of inhibiting their growth [29]. Anaplerosis via PC has received much attention in the past few years. Fan *et al*. [14] have reported a high rate of pyruvate carboxylation flux as a result of overexpression of PC protein in non-small cell lung cancer, suggesting that this cancer is highly anabolic. Overexpression of PC in this type of cancer indicates its need to fulfill the high anabolic demands during the establishment of the primary location [14]. Cheng *et al* [19] have also found that PC is overexpressed in glioblastomas in which PC is required to support tumor growth. Subsequent studies revealed that the presence of PC activity in glioblastomas is consistent with its anaplerotic role [30]. Furthermore, pyruvate carboxylation is also shown to be an important anaplerotic reaction that contributes to the citrate pool during reductive carboxylation in osteosarcoma and renal carcinoma cell lines [31].

Here we show that PC is also highly expressed in breast cancer tissue but not in the normal breast tissue. The statistical association between the levels of PC expression and the tumor size and stage prompted us to investigate the role of this enzyme in supporting growth and invasion. That expression of PC was highly abundant in two aggressive breast cancer cell lines, i.e. MDA-MB-231 and MDA-MB-435 but low in less aggressive cell lines, i.e. MCF-7 and SKBR3 hints at the involvement of this enzyme in the aggressive phenotype of these two metastatic cell lines. Suppression of PC expression in both MDA-MB-231 and MDA-MB-435 cells retarded cell proliferation rate, suggesting that these cell lines modestly depend on anaplerosis via the PC reaction. The necessity for PC in supporting growth of MDA-MB-231 cells appears to be different from the SF-XL glioblastoma cell line which utilizes glutaminolysis rather than pyruvate carboxylation as the primary anaplerotic reaction [32] because suppression of PC expression does not affect its growth under glutamine-dependent conditions [15]. However, under glutamine-depleted growth conditions, the PC-knockdown SF-XL glioblastoma cell line shows marked reduction (>80%) of proliferation [15], indicating that the glioblastoma cell line uses pyruvate carboxylation as an alternative route to support its growth during glutamine-depleted conditions. However this is not the case for MDA-MB-231 and MDA-MB-435 cells because suppression of PC expression still allows them to grow, albeit at the 50% reduced proliferation rate observed under glutamine-dependent growth conditions. A similar but not identical phenotype of the PC-knockdown MDA-MB-231 and MDA-MB-435 cells was observed under glutamine-independent growth conditions. An involvement of PC in the growth phenotype of MDA-MB-231 cells is also consistent with the association between the levels of PC expression and sizes of breast tumor in clinical samples. Low expression levels of PC in breast tumors with a smaller size of tumor (< 4 cm^3^) may limit their growth compared with the larger size of tumors which contain higher levels of PC expression (P < 0.05) [Table 1]. The above observations were also confirmed by overexpression of PC in MCF-7, which is a low metastatic cell line. MCF-7 cells overexpressing PC show enhanced growth rate, motility and invasion. The enhanced growth phenotype of MCF-7 with overexpressed PC was consistent with previous reports which show that ectopic expression of PC in many mammalian cell lines enhances cell growth and biomass production [33,34,35].

Although several studies have pointed to the importance of PC in providing oxaloacetate which is the first TCA cycle intermediate, that in turn is converted to other precursor molecules such as citrate required for lipid and nucleic acid synthesis for rapid tumor growth, not much is known as to whether PC is required to support migration and invasion ability of some tumors [14, 15, 19]. Our present study is the first report to show a strong association between the levels of PC expression in breast tissues of patients and stages of cancer progression (P < 0.05), which suggests PC is involved in metastasis. This was also studied in MDA-MB-231 and MDA-MB-435 cells. Suppression of expression of PC markedly reduced both migration and invasion ability through an extracellular matrix under both glutamine-nourished and glutamine-depleted conditions, suggesting that PC is required to support these aggressive phenotypes of MDA-MB-231 cells. The negative staining of PC expression by immunohistochemistry in the normal area of breast tissue is consistent with the low level of expression of PC in the MCF-7 cell line. The transcriptional mechanism underlying overexpression of PC in MDA-MB-231 over MCF-7 is due to the selective activation of the distal rather than the proximal promoter of the human PC gene (Fig 2C). The specific transcription of the distal rather than the proximal promoter of the human PC gene may be attributed to the presence of different putative transcription factor binding sites of both promoters [24] during the transition of a low to a high metastatic phenotype. Interestingly, Lee *et al*. (2012) have shown that the MCF-7 cells stimulated with Wnt 1 or Wnt3a ligands, or ectopically expressed with Snail, a target transcription factor of Wnt signaling, show a marked increase in PC expression. Because the Wnt signaling pathway also induces Snail-dependent epithelial-mesenchymal transition (EMT), which is responsible for invasion and metastasis in many tumors [36,37], the authors suggest that anaplerosis via up-regulation of PC expression is one of several metabolic responses of breast tumor during EMT [38]. Although this study underscores that the Wnt signaling pathway is important for transcriptional induction of the PC gene, the authors cannot detect direct binding of Snail, an effector transcription factor in response to Wnt signaling to the distal promoter of human PC gene [38].

Although the involvement of PC with migration and *in vitro* invasion of MDA-MB-231 cells *per se* is yet to be elucidated, it is apparent that migration of the tumors from the primary location to the distal organs during metastasis requires multiple biological steps such as intravasation into the circulation, followed by migration, extravasation to a second site for adhesion, tumorigenesis and angiogenesis [39], all of which require substantial amounts of ATP to provide a source of energy [40]. This is consistent with the findings that metastasized tumors including breast and osteosarcoma show up-regulation of energy generating pathways including TCA cycle activity and nucleotide metabolism [40,41,42]. As PC provides oxaloacetate, the first intermediate in the TCA cycle, disruption of oxaloacetate supply by suppressing PC expression may disrupt the continual supply of this intermediate required for oxidation of pyruvate hence lowering ATP production required to support cell movement. Although many tumors are highly glycolytic, growing evidence have shown that highly metastasized tumors also utilize mitochondria to oxidize glucose. In ovarian cancer and possibly breast cancer [43,44], the metastasized tumor invades the adjacent adipose tissue to hijack lipids to use as an alternative fuel apart from glucose to support tumor growth and invasion. Since β-oxidation of fatty acids produces large amounts of acetyl-CoA which is subsequently oxidized via the condensation with oxaloacetate in the TCA cycle, a high abundance of PC would ensure that oxaloacetate is not limited during β-oxidation of the hijacked lipids. The presence of PC may be involved in gluconeogenesis. Leithner *et al* [45] have recently shown that lung cancer utilizes gluconeogenesis to recycle lactate back to glucose as an adaptive metabolic response to glucose depletion. This was born out by the observation of overexpression of mitochondrial phosphoenolpyruvate carboxykinase (PEPCK-M), one of four gluconeogenic enzymes concomitant with the increased flux of lactate to phosphoenolpyruvate. However, the level of PC expression varied in the three lung cancers examined in this study and was not up-regulated by low glucose concentrations.

Other possible roles of PC in supporting breast cancer growth and metastasis may be linked to the metabolic coupling factor, NADPH. In pancreatic β-cells, PC is highly abundant to support the high activity of pyruvate cycling which provides a large amount of NADPH, required for glucose-induced insulin secretion [46]. In tumors, it is possible that the similar pyruvate/malate shuttle [47,48,49] may operate in the similar manner as in the pancreatic islet. Several lines of evidence have suggested that NADPH may be a key molecule which links between reactive oxygen species (ROS) formation and the EMT via the overexpression of NADPH oxidase, notably the NOX4 isoform which is overexpressed in many tumors [50,51]. NOX4 is known to mediate the production of ROS which triggers cellular senescence, resistance to apoptosis and tumorigenic transformation [52].

The lack of statistical association between expression of PC and ER as well as PR and HER2 in cancer breast tissues indicates that PC is not regulated by these hormones or growth factor. This is also consistent with the phenotypes of MCF-7 and MDA-MB-231 cells which are ER^+^ and ER^-^, respectively [53].

In summary we have shown that PC is expressed in breast cancer at a higher level than in the normal breast tissue, and exhibits a statistical association with tumor size and the progression of metastasis. Using the highly metastatic breast cancer cell lines, MDA-MB-231 and MDA-MB-435 as models, we showed that suppression of PC expression markedly reduces the proliferation, migration and *in vitro* invasion ability of cells, highlighting the possible roles of PC in supporting these processes during oncogenesis and progression of breast cancer.
